# Estimation of Autoregressive Parameters from Noisy Observations Using Iterated Covariance Updates

**DOI:** 10.3390/e22050572

**Published:** 2020-05-19

**Authors:** Todd K. Moon, Jacob H. Gunther

**Affiliations:** Electrical and Computer Engineering Department, Utah State University, Logan, UT 84332, USA; jake.gunther@usu.edu

**Keywords:** autoregressive model estimation, spectrum estimation, vector AR model, RLS algorithm

## Abstract

Estimating the parameters of the autoregressive (AR) random process is a problem that has been well-studied. In many applications, only noisy measurements of AR process are available. The effect of the additive noise is that the system can be modeled as an AR model with colored noise, even when the measurement noise is white, where the correlation matrix depends on the AR parameters. Because of the correlation, it is expedient to compute using multiple stacked observations. Performing a weighted least-squares estimation of the AR parameters using an inverse covariance weighting can provide significantly better parameter estimates, with improvement increasing with the stack depth. The estimation algorithm is essentially a vector RLS adaptive filter, with time-varying covariance matrix. Different ways of estimating the unknown covariance are presented, as well as a method to estimate the variances of the AR and observation noise. The notation is extended to vector autoregressive (VAR) processes. Simulation results demonstrate performance improvements in coefficient error and in spectrum estimation.

## 1. Introduction

The problem of estimating the parameters $\left\{b_{i},i=1,\ldots ,p\right\}$ of an autoregressive (AR) process

(1) $$\xi \left(m\right)=\sum_{i=1}^{p}b_{i}\xi \left(m-i\right)+\eta \left(m\right),$$

where the input η(n) is a white noise process, is important in many aspects of signal processing. It plays roles a variety of applications, such as speech coding and analysis (see References [1,2,3,4,5,6,7]). Autoregressive modeling is instrumental in many spectrum estimation algorithms, and algorithms for noise-free measurements have been developed from that perspective [8,9,10,11,12,13,14]; see also Reference [5] for a survey and perspective. Vector autoregressive modeling is also important in econometric modeling [15]. Among many other applications, AR models have also been used in biomedical signal processing (see, e.g., Reference [16]); communication (see, e.g., Reference [17]); and financial modeling (see, e.g., Reference [15]).

Because of its importance, the AR parameter estimation problem has been well-studied from a realization of noise-free process {ξ(m)} When autocorrelations $r_{k}=E\left[\xi \left(m\right)\xi^{*}\left(m-k\right)\right]$ are known (or estimated), the Yule-Walker equations describe the solutions [18,19]. This problem may also be considered as the problem of finding optimal predictor coefficients, and solutions invoking adaptive filters are well known [20]. The parameters can also be estimated using a Kalman filter applied to a statespace formulation ([21], Section 3.3), in which the unknown parameters are taken to be the state, and the observation matrix is from the measurements. The Kalman filter method bears resemblance to the approach presented here, but as will be shown there are differences between the two. First, in the case of noisy observations, the previous AR values ξ(m−i),i=1,…,p are not known, so the elements of observation matrix are only approximately known in the Kalman filter approach. No such approximation is used in the method here. Second, the approach presented here does not require the introduction of the system noise with the question of what the variance should be. And thirdly, instead of a scalar observation equation, vector observations are used, allowing the covariance structure to be used with a time-varying covariance.

It is well known ([5], Section 6.7), [22,23] that additive noise broadens spectral peaks in autoregressive spectral estimation and can result in loss of resolution of closely-spaced sinusoids. Dealing with noise added to AR processes has been addressed many times in the literature, as we summarize below. None of these previous methods use the covariance structure employed here. Some of these techniques are related to finding rank *p* representations of a data matrix (using, for example, an SVD or total least squares) [24,25,26,27,28,29,30]. From another perspective, it is known that white noise added to an AR(p) process produces an ARMA(p,p) process. This has resulted in algorithms for dealing with the noisy AR process using an ARMA estimator. For example, modified Yule-Walker (YW) equations [31] can be used to find the ARMA model. However, this does not take advantage of the relationship between the AR and the MA parameters in this noisy AR case. Another approach is to estimate the ARMA process using a large-order AR model. Another approach to handling noisy AR observations is total least squares (TLS) [32,33,34,35]. None of these approaches is equivalent to the approach described here. Noisy AR estimation has been studied over many years by Zheng [36,37,38,39,40,41,42,43,44,45,46,47,48,49,50,51,52,53,54,55], who estimates the variance of the observation noise to reduce bias. An approach that is somewhat similar to our approach is bias-corrected RLC (BCRLS) [56,57,58,59,60,61], but the BRCLS does not employ the covariance matrix-weighted least squares used here. Other approaches to noisy AR parameter estimation have been investigated in References [4,62,63,64,65,66,67,68,69,70,71,72,73,74,75,76,77,78,79,80,81,82,83,84,85,86,87,88,89,90,91,92]

The approach described here uses the fact that the observation noise produces an AR-like process with correlated noise, where the noise correlation depends on the AR coefficients. The correlation is exploited by dealing with vectors of stacked observations, rather than scalar observations. A maximum likelihood approach results in a weighted least-squares problem, in which previous estimates are used to update the covariance matrix in an iterative fashion. We refer to this general algorithm as iterative covariance-weighted autoregressive estimation (ICWARE). While maximum likelihood has been used for AR parameter estimation [18] ([93], Chapter 5), [94], in previous estimators the noise is assumed white, so the autocorrelation structure derived here is not exploited. Iterative parameter estimation has been used for system parameter estimation. An early iterative method of fitting a given impulse response to a transfer function, without an explicit noise model, is in Reference [95]. Iterative quadratic ML (IQML) [96] is another iterative technique. IQML is similar to the algorithm of Reference [97], which requires knowledge of the input signal. IQML is also similar to the method of Reference [98]. An IQML approach [99] has been used for noisy AR estimation. None of these make use of the covariance weighting of ICWARE.

There is some similarity of this approach to the measurement error models studied in the statistical literature [100], in which parameters of regression models such as $Y_{t}=\beta_{0}+\beta_{1}x_{t}+e_{t}$ are estimated when only noisy observations of the regressors $X_{t}=x_{t}+u_{t}$ are available. These noisy observations induce changes to conventional minimum mean-square error (MMSE) estimators which are somewhat related to errors-in-variables approaches to AR estimation [101,102,103].

Another approach to this problem makes use of coupled Kalman filters [104] or cross-coupled $H_{\infty }$ filters [105,106] has been developed. In essence these move beyond the Kalman filter approach referenced above ([21], Section 3.3) and operate as follows. One Kalman filter estimates the true autoregressive value $\hat{\zeta }\left(m\right)$, assuming knowledge of the AR parameters $\left\{b_{i}\right\}$, while the other Kalman filter estimates the $\left\{b_{i}\right\}$ using the estimates $\hat{\zeta }\left(m\right)$ as if they were the true values. These two filters operate in conjunction to jointly converge to the AR parameter estimate. To evaluate our method in comparison with the dual Kalman filter method, the algorithm here is compare to models also seen in Reference [104].

Recently, Monte Carlo methods, such as particle filtering has been used for AR and ARMA estimation [107,108,109,110]. Particle filtering methods are quite general and can track dynamical variables and jointly estimate static parameters. These methods offer the possibility of convergence to good estimates in situations when the noise is not Gaussian (which has been assumed in this paper), but offer the usual drawbacks of potentially high computational complexity if many particles are used. These represent an alternative to the methods of this paper, and a thorough comparison, while valuable, lies beyond the scope of this paper.

This topic is relevant to its journal of publication (*Entropy*), since spectral analysis under a maximum entropy criterion has (famously) been shown to be equivalent to AR modeling [11]. As this work shows, however, when there are noisy observations particular attention must be devoted to the information present in the autocorrelation matrix of the observations, beyond the first order equations that result from the historical maximum entropy approach.

We present the method first for the scalar AR model. For generality, noise is assumed to be circular complex Gaussian noise; modifications for real signals is straightforward. In addition to the coefficient estimation problem, we also address the problem of variance estimation. Following development for scalar AR processes, the notation is developed for the vector AR (VAR) processes. Several simulation results demonstrate that the ICWARE method provide significant improvements over classical YW-type methods.

## 2. Scalar Parameter Estimation in Noise

**Figure 1:**
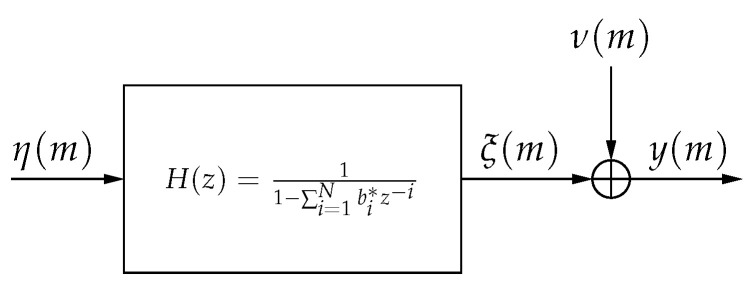
Noisy Autoregressive Model.

The noisy observation equation, represented by the system in Figure 1, is

(2) $$y\left(m\right)=\xi \left(m\right)+\nu \left(m\right)=\sum_{i=1}^{p}b_{i}\xi \left(m-i\right)+\eta \left(m\right)+\nu \left(m\right),$$

where ξ(m)∈C and each $b_{i}\in C$, and where ν(m) is a white noise process. The process {ξ(m)} is said to be the noise-free AR process, and the process {y(m)} is said to be the noisy AR process.

The input and observation noises are assumed to be complex circular Gaussian, so that

$$\eta \left(m\right)=\sigma_{\eta }\left(N\left(0,1\right)+jN\left(0,1\right)\right)\;\mathrm{and}\;\nu \left(m\right)=\sigma_{\nu }\left(N\left(0,1\right)+jN\left(0,1\right)\right),$$

where the real and imaginary parts are uncorrelated, and draws at different times are independent. Furthermore, these noises are assumed to be uncorrelated with each other, so

$$E\left[\nu \left(m\right)\nu {\left(m-l\right)}^{*}\right]=2\sigma_{\nu }^{2}\delta_{l}\;E\left[\eta \left(m\right)\eta {\left(m-l\right)}^{*}\right]=2\sigma_{\eta }^{2}\delta_{l}\;E\left[\nu \left(m\right)\eta \left(n^{*}\right)\right]=0.$$

Let $b={\left[\begin{array}{cccc} b_{1} & b_{2} & \cdots & b_{p} \end{array}\right]}^{T}$ and $y\left(i\right)={\left[\begin{array}{cccc} y(i) & y(i-1) & \cdots & y(i-p+1) \end{array}\right]}^{T}$ (this vector is re-defined below to have a different length). The conventional least-squares approach to estimating b (in the forward prediction error sense) would be to compute

(3) $$\hat{b}=\arg\min_{b}\sum_{i}{\left|y\left(i\right)-b^{T}y\left(i-1\right)\right|}^{2},$$

which results in the conventional estimate

$${\hat{b}}^{*}={\left(\sum_{i}y\left(i-1\right)y{\left(i-1\right)}^{H}\right)}^{-1}\left(\sum_{i}y^{*}\left(i\right)y\left(i-1\right)\right).$$

This is essentially the covariance method [19]. The estimate (3) can be computed using recursive formulation, resulting in a recursive least-squares (RLS) adaptive filter. This approach essentially neglects the noise ν(m). It is known that the noise will bias the estimate results [22].

Note: The error could also be computed in a backward prediction error sense, or in a combined forward-backward sense as is done in some spectrum estimation algorithms such as the modified covariance method [14], ([5], Section 75). The ICWARE method we describe below can be extended to these generalizations as well, but for brevity only forward prediction error is used.

Substituting the AR signal in terms of the observation ξ(m)=y(m)−ν(m) into the AR model (1) and using the observation model (2) we obtain

(4)y(m)=∑i=1pbiy(m−i)+(η(m)+ν(m)−∑i=1pbiν(m−i))(5)=∑i=1pbiy(m−i)+w(m),

where

$$w\left(m\right)=\left(\eta \left(m\right)+\nu \left(m\right)-\sum_{i=1}^{p}b_{i}\nu \left(m-i\right)\right)$$

denotes the noise. The expression (4) is an ARMA(p,p) process and is the basis for some ARMA-based approaches to AR estimation in noise. The expression (5) has the appearance of an AR(p) process, except that the noise w(m) is not white, having correlation

(6) $$\begin{array}{c} E\left[w\left(m\right)w^{*}\left(m\right)\right]=E\left[\left(\eta \left(m\right)+\nu \left(m\right)-\sum_{i=1}^{p}b_{i}\nu \left(m-i\right)\right)\right.\left.{\left(\eta \left(m\right)+\nu \left(m\right)-\sum_{i=1}^{p}b_{i}\nu \left(m-i\right)\right)}^{*}\right]\\ \;\;=2(\sigma_{\eta }^{2}+\sigma_{\nu }^{2}+\sum_{i=1}^{p}|b_{i}{|}^{2}\sigma_{\nu }^{2})\overset{▵}{=}r_{w}\left(0\right)\\ E\left[w\left(m\right)w{\left(m+\ell \right)}^{*}\right]=E\left[\left(\eta \left(m\right)+\nu \left(m\right)-\sum_{i=1}^{p}b_{i}\nu \left(m-i\right)\right)\right.\left.{\left(\eta \left(m\;+\;\ell \right)\;+\;\;\nu \left(m\;+\;\ell \right)\;-\;\sum_{i=1}^{p}b_{i}\nu \left(m\;+\;\ell \;-\;i\right)\right)}^{*}\right]\\ \;\;=2\sigma_{\nu }^{2}\left(\sum_{i=1}^{p-\ell }b_{i}b_{i+\ell }^{*}-b_{\ell }^{*}\right)\overset{▵}{=}r_{w}\left(-\ell \right). \end{array}$$

If the noise w(n) were uncorrelated, the solution of (3) would be least-squares optimal. However, since w(m) is correlated, the sample-by-sample approach of (3) is suboptimal since it does not take the correlation into account. As we now show, there is information in the covariance structure of the signal that can be used to improve the estimate. To take the correlation into account, stack the observations of (5) into vectors of length *d* (that is, the depth), as

(7) $$\begin{array}{cc} \; & \left[\begin{array}{c} y(m)\\ y(m-1)\\ \vdots \\ y(m-d+1) \end{array}\right]=\left[\begin{array}{ccc} y(m-1) & \cdots & y(m-p)\\ y(m-2) & \cdots & y(m-1-p)\\ \vdots \\ y(m-d) & \cdots & y(m-d-p+1) \end{array}\right]\left[\begin{array}{c} b_{1}\\ b_{2}\\ \vdots \\ b_{p} \end{array}\right]+\left[\begin{array}{c} w(m)\\ w(m-1)\\ \vdots \\ w(m-d+1) \end{array}\right] \end{array}.$$

Write this as

y(m)=Y(m)b+w(m)

The correlation matrix of the vector noise is

(8) $$\begin{array}{cc} \; & E[w\left(m\right)w{\left(m\right)}^{H}]=\\ \; & \;\left[\begin{array}{cccc} r_{w}\left(0\right) & r_{w}\left(-1\right) & \ldots & r_{w}\left(-\left(d-1\right)\right)\\ r_{w}\left(1\right) & r_{w}\left(0\right) & \ldots & r_{w}\left(-\left(d-2\right)\right)\\ \vdots \\ r_{w}\left(d-1\right) & r_{w}\left(d-2\right) & \cdots & r_{w}\left(0\right) \end{array}\right]\overset{▵}{=}R_{w}\left(b\right). \end{array}$$

In moving through a sequence of data, the data can be advanced by a skip *s* to form a sequence of vectors y(m),y(m+s),y(m+2s),…,y(m+s(k−1)) and matrices Y(m),Y(m+s),Y(m+2s),…,Y(m+s(k−1)), which we write for convenience as $y_{0},y_{1},\ldots ,y_{k-1}$, and $Y_{0},Y_{1},\ldots ,Y_{k-1}$, respectively. The index *m* is chosen to make it possible to use only causal data, m≥d+p−1.

Assuming independence (such as when s≥d) and assuming that the correlation function $R_{w}\left(b\right)$ is known, the likelihood function can be written as a circular complex Gaussian as

(9) $$\begin{array}{cc} \; & f\left(y_{0},\ldots ,y_{k-1}|b,R_{w}\left(b\right)\right)\propto \frac{1}{|\det\left(R_{w}\left(b\right)\right){|}^{k}}\exp\left[-\sum_{i=0}^{k-1}{\left(y_{i}-Y_{i}b\right)}^{H}R_{w}{\left(b\right)}^{-1}\left(y_{i}-Y_{i}b\right)\right] \end{array}.$$

Finding the true maximum likelihood solution by directly maximizing (9) with respect to b is difficult, since b enters nonlinearly in $R_{w}\left(b\right)$. Instead, an iterative approach is employed. An estimated value $R_{w}{\left(b\right)}_{i}$ is used at the *i*th step. Assuming still that each $R_{w}{\left(b\right)}_{i}$ is known, a maximum likelihood solution may be obtained from

(10) $${\hat{b}}_{k}=\arg\min_{b}\sum_{i=1}^{k}\lambda^{k-i}{∥y_{i}-Y_{i}b∥}_{R_{w}{\left(b\right)}_{i}^{-1}}^{2}.$$

Here, a forgetting factor $\lambda^{k-i}$, with λ<1, has been introduced to allow for tracking a time-varying b. Significantly, the norm here is weighted by the inverse covariance $R_{w}{\left(b\right)}_{i}^{-1}$. If we take d=1, we obtain the regular least-squares problem, and the additional correlation structure does not appear. Taking the gradient of the cost functional (10) results in the normal equation

(11) $$\begin{array}{cc} \; & \left(\sum_{i=1}^{k}\lambda^{k-i}Y_{i}^{H}R_{w}{\left(b\right)}_{i}^{-1}Y_{i}\right){\hat{b}}_{k}=\left(\sum_{i=1}^{k}\lambda^{k-i}Y_{i}^{H}R_{w}{\left(b\right)}_{i}^{-1}y_{i}\right). \end{array}$$

Write this as $\Phi_{k}^{-1}{\hat{b}}_{k}=\phi_{k}$. This can be recursively updated using RLS recursions (see, e.g., References ([111], Section 8.7), [112]), starting from an initial $\Phi_{-1}=\frac{1}{\delta }I$ for a small scalar δ and propagating $\Phi_{k}$.

Computing (11) requires knowledge of $R_{w}{\left(b\right)}_{i}$, which is not available since b is to be found. In combination with a recursively updated solution to (11), $R_{w}{\left(b\right)}_{i}$ is estimated using previously computed values of $\hat{b}$ and used as a time-varying covariance matrix. That is, we write $R_{w}{\left(b\right)}_{i}=R_{w}{\left({\hat{b}}_{i-1}\right)}_{i}$. The result is the framework shown in Algorithm 1, similar to a vector RLS adaptive filter.
**Algorithm 1:** AR Parameter estimation with noisy data.Input: $y_{k}$, $Y_{k}$, $R_{w}{\left({\hat{b}}_{k-1}\right)}_{k}$Previous Conditions: $\Phi_{k-1}$, ${\hat{b}}_{k-1}$Compute
$$\begin{array}{cc} K_{k} & =\lambda^{-1}\Phi_{k-1}Y_{k}^{H}{\left(\lambda^{-1}Y_{k}\Phi_{k-1}Y_{k}^{H}+R_{w}\left({\hat{b}}_{k-1}\right)\right)}^{-1}\\ {\hat{b}}_{k} & ={\hat{b}}_{k-1}+K_{k}\left(y_{k}-Y_{k}{\hat{b}}_{k-1}\right)\\ \Phi_{k} & =\lambda^{-1}\left(I-\lambda^{-1}K_{k}Y_{k}\right)\Phi_{k-1}. \end{array}$$Update the covariance to obtain $R_{w}{\left({\hat{b}}_{k}\right)}_{k+1}$ and compute $R_{w}{\left({\hat{b}}_{k}\right)}_{k+1}^{-1}$.Return ${\hat{b}}_{k}$, $\Phi_{k}$, $R{\left({\hat{b}}_{k}\right)}_{k+1}$.

The “Update the covariance” step, detailed below, is how the structure of $R_{b}\left(b\right)$ can be used to improve the parameter estimates.

We have explored several different approaches to computing and using $R_{w}\left(\hat{b}\right)$:

1. Ignore $R_{w}\left(b\right)$: Neglect the correlation structure and simply assume that $R_{w}{\left({\hat{b}}_{k}\right)}_{i}=I$. This gives the equivalent of taking a scalar measurement and is used as a sort of worst-case basis for comparison among the different algorithms.
2. Use the correct value of $R_{w}\left(b\right)$: That is, assume that b and $\sigma_{\nu }^{2}$ and $\sigma_{\eta }^{2}$ are known and compute $R_{w}{\left(\hat{b}\right)}_{k}$ according to (6). This provides a limit on best-case performance against which other methods can be compared.
3. Use the estimate of b: Using the correct values of $\sigma_{\nu }^{2}$ and $\sigma_{\eta }^{2}$, compute the autocorrelation matrix using ${\hat{b}}_{k}$ in (6).
4. Estimate $\hat{b}$, fix $\sigma_{\nu }^{2}$ and $\sigma_{\eta }^{2}$: With assumed values of $\sigma_{\nu }^{2}$ and $\sigma_{\eta }^{2}$, compute the autocorrelation matrix using ${\hat{b}}_{k}$ in (6).
5. Estimate everything: Estimate the values of $\sigma_{\nu }^{2}$ and $\sigma_{\eta }^{2}$, then use them with ${\hat{b}}_{k}$ in (6).

In the early stages while ${\hat{b}}_{k}$ is poorly converged, it is best to use option 1 (assuming identity covariance matrix) until $\hat{b}$ has settled near its final value, then switch to option 4 using estimated ${\hat{b}}_{k}$ until the moments in Σ(b) have converged sufficiently well that reliable estimates of the variances can be obtained and option 5 can be used. As described in Section 3, the information necessary to estimate the variances can be accumulated without yet having a decent estimate for ${\hat{b}}_{k}$.

The covariance update/inverse does not necessarily need to be done at every step. Particularly as ${\hat{b}}_{k}$ settles towards its final value, there is little to be gained by updating $R_{w}\left(\hat{b}\right)$ at every step.

## 3. Estimating the Variances

As the results below will indicate, fixed values of $\sigma_{\nu }^{2}$ and $\sigma_{\eta }^{2}$ can be used in the estimate of $R_{w}\left(b\right)$ instead of estimated values, so for the purposes of estimating b, it is not strictly necessary to estimate these variances. But for other purposes it may be necessary to have an estimate of $\sigma_{\nu }^{2}$ and $\sigma_{\eta }^{2}$. A maximum likelihood estimation approach is thus described in this section.

For a given estimate of the coefficients $\hat{b}$, write $R_{w}\left(\hat{b}\right)$ as

$$R_{w}\left(\hat{b}\right)=2\sigma_{\eta }^{2}I+2\sigma_{\nu }^{2}B\left(\hat{b}\right).$$

For a sequence of *k* vectors $y_{0},y_{1},\ldots ,y_{k-1}$, assumed to be nonoverlapping, encompassing *N* samples, the joint log likelihood function is a complex (circularly symmetric) Gaussian

(12) $$\begin{array}{c} \log f\left(y_{0},y_{1},\ldots ,y_{k-1}|b,R_{w}\left(b\right),\sigma_{\nu }^{2},\sigma_{\eta }^{2}\right)=-N\log\pi -k\log\det R_{w}\left(b\right)-\sum_{i=0}^{k-1}{\left(y_{i}-Y_{i}b\right)}^{H}R_{w}{\left(b\right)}^{-1}\left(y_{i}-Y_{i}b\right)]. \end{array}$$

Let

(13) $$\Sigma \left(b\right)=\frac{1}{k}\sum_{i=0}^{k-1}\left(y_{i}-Y_{i}b\right){\left(y_{i}-Y_{i}b\right)}^{H}$$

denote the sample covariance. It can be shown that (see Appendix A)

(14) $$\begin{array}{cc} \; & \frac{\partial }{\partial \sigma_{\eta }^{2}}\log f\left(y_{1},y_{2},\ldots ,y_{k}|b,\sigma_{\nu }^{2},\sigma_{\eta }^{2}\right)=-k\mathrm{tr}\left(R_{w}{\left(b\right)}^{-1}\right)+k\mathrm{tr}(R_{w}{\left(b\right)}^{-1}\Sigma \left(b\right)R_{w}{\left(b\right)}^{-1}. \end{array}$$

and that

(15) $$\begin{array}{c} \frac{\partial }{\partial \sigma_{\nu }^{2}}\log f\left(y_{1},y_{2},\ldots ,y_{k}|b,\sigma_{\nu }^{2},\sigma_{\eta }^{2}\right)=-k\sum_{i,j}{\left(R_{w}{\left(b\right)}^{-1}\right)}_{i,j}{\left(B\left(b\right)\right)}_{ij}+k\sum_{i,j}{\left(R_{w}{\left(b\right)}^{-1}\Sigma \left(b\right)R_{w}{\left(b\right)}^{-1}\right)}_{i,j}{\left(B\left(b\right)\right)}_{i,j}. \end{array}$$

The gradients (14) and (15) are equal to zero when

(16) $$R_{w}\left(b\right)=\Sigma \left(b\right).$$

The variance estimates are chosen to satisfy the condition (16) using an estimate of b from previous estimates, that is

(17) $$2\sigma_{\eta }^{2}I+2\sigma_{\nu }^{2}B\left(\hat{b}\right)=\Sigma \left(\hat{b}\right).$$

We define the offset trace as

$$\mathrm{tr}\left(B,\ell \right)=\sum_{i}B_{i,i+\ell },$$

where the usual trace is obtained when ℓ=0, and for *ℓ* > 0, the sum is taken on the *ℓ*th superdiagonal. Let $\beta_{\ell }=\mathrm{tr}\left(B\left(\hat{b}\right),\ell \right)$ for ℓ=0,1,…,d−1, where that is, the trace on the *ℓ*th superdiagonal. Taking the offset trace of (17) gives

$$\begin{array}{cc} 2d\sigma_{\eta }^{2}+2\sigma_{\nu }^{2}\beta_{0} & =\mathrm{tr}(\Sigma \left(\hat{b}\right),0)\\ 2\sigma_{\nu }^{2}\beta_{\ell } & =\mathrm{tr}(\Sigma \left(\hat{b}\right),\ell ),\;\ell =1,2,\ldots ,d-1. \end{array}$$

The ML estimates of the variances are the solutions to

(18) $$2\left[\begin{array}{cc} d & \beta_{0}\\ 0 & \beta_{1}\\ \vdots \\ 0 & \beta_{d-1} \end{array}\right]\left[\begin{array}{c} {\hat{\sigma }}_{\eta }^{2}\\ {\hat{\sigma }}_{\nu }^{2} \end{array}\right]=\left[\begin{array}{c} \mathrm{tr}(\Sigma \left(\hat{b}\right),0)\\ \mathrm{tr}(\Sigma \left(\hat{b}\right),1)\\ \vdots \\ \mathrm{tr}(\Sigma \left(\hat{b}\right),d-1). \end{array}\right]$$

A significant number of terms of $\Sigma (\hat{b})$ must be accumulated in order to estimate the variances well. Initially, before reasonably accurate estimates of b are available, using inaccurate $\hat{b}$s can result in a highly inaccurate $\Sigma (\hat{b})$. As a result, it is desirable to accumulate moments of $y_{i}$ and $Y_{i}$ without using $\hat{b}$. The trace of the sample covariance is

$$\begin{array}{c} \mathrm{tr}\left(\Sigma \left(b\right),\ell \right)=\frac{1}{k}\sum_{i=1}^{k}\mathrm{tr}\left(y_{i}y_{i}^{H},\ell \right)-\mathrm{tr}\left(y_{i}b^{H}Y_{i}^{H},\ell \right)-\mathrm{tr}\left(Y_{i}by_{i}^{H},\ell \right)+\mathrm{tr}\left(Y_{i}bb^{H}Y_{i}^{H},\ell \right). \end{array}$$

The different terms can be written as

$$\begin{array}{cc} \sum_{i}\mathrm{tr}\left(y_{i}b^{H}Y_{i}^{H},\ell \right) & =\sum_{j}\sum_{k}\sum_{i}y_{i,j}b_{k}^{*}Y_{i,j+\ell ,k}^{*}=\sum_{k}b_{k}^{*}\sum_{i}{\left[Y_{i,\ell +1:\mathrm{end},k}\right]}^{H}y_{i,1:\mathrm{end}-\ell } \end{array}$$

$$\begin{array}{cc} \sum_{i}\mathrm{tr}\left(Y_{i}by_{i}^{H}\right) & =\sum_{i}\sum_{j}\sum_{k}Y_{i,k,j}b_{j}y_{i,k+\ell }^{*}=\sum_{j}b_{j}\sum_{i}{\left[y_{i,1+\ell :\mathrm{end}}\right]}^{H}Y_{i,1:\mathrm{end}-\ell ,j}. \end{array}$$

(It is noted that, except when ℓ=0, these two terms are not conjugates of each other, so that both of these terms must be retained.)

$$\begin{array}{c} \sum_{i}\mathrm{tr}\left(Y_{i}bb^{H}Y_{i}^{H},\ell \right)=\sum_{j}\sum_{k}\sum_{l}\sum_{i}Y_{i,k,j}{\left(bb^{H}\right)}_{j,l}{\left[Y_{i}^{H}\right]}_{l,k+\ell }\\ \;=\sum_{j}\sum_{l}{\left(bb^{H}\right)}_{j,l}\sum_{i}{\left[Y_{i,1+\ell :\mathrm{end},:}\right]}^{H}Y_{i,1:\mathrm{end}-\ell ,:} \end{array}$$

These terms can be computed by recursively propagating the following quantities, for k=1,2,…,p and ℓ=0,1,…,p:

$$\begin{array}{cc} a^{0}\left(\ell ,k\right) & =\frac{1}{k}\sum_{i=0}^{k-1}y_{i,1+\ell ,\mathrm{end}}^{H}y_{i,1:\mathrm{end}-\ell }\\ & =\frac{1}{k}\left(y_{k,1\;+\;\ell :\mathrm{end}}^{H}y_{k,1:\mathrm{end}-\ell }\;+\;\left(k\;-\;1\right)a^{0}\left(\ell ,k\;-\;1\right)\right)\\ a_{k}^{1}\left(\ell ,k\right) & =\frac{1}{k}\sum_{i=0}^{k-1}{\left[Y_{i,\ell +1:\mathrm{end},k}\right]}^{H}y_{i,1:\mathrm{end}-\ell }\\ & =\frac{1}{k}\left({\left[Y_{k,\ell +1:\mathrm{end},k}\right]}^{H}y_{k,1:\mathrm{end}-\ell }+\left(k-1\right)a_{k}^{1}\left(\ell ,k-1\right)\right)\\ a_{k}^{2}\left(\ell ,k\right) & =\frac{1}{k}\sum_{i=0}^{k-1}y_{i,\ell \;+\;1:\mathrm{end}}^{H}Y_{i,1:\mathrm{end}\;-\;\ell ,k}\\ & =\frac{1}{k}\left(y_{k,\ell \;+\;1:\mathrm{end}}^{H}Y_{k,1:\mathrm{end}\;-\;\ell ,k}\;+\;\left(k\;-\;1\right)a_{k}^{2}\left(\ell ,k\;-\;1\right)\right)\\ A(\ell ,k) & =\frac{1}{k}\sum_{i=0}^{k-1}Y_{i,\ell +1:\mathrm{end},:}^{H}Y_{i,1:\mathrm{end}-\ell ,:}\\ & =\frac{1}{k}\left(Y_{k,\ell \;+\;1:\mathrm{end},:}^{H}Y_{k,1:\mathrm{end}\;-\;\ell ,:}\;+\;\left(k\;-\;1\right)A\left(\ell ,k\;-\;1\right)\right). \end{array}$$

Then

$$\begin{array}{cc} \mathrm{tr}(\Sigma \left(\hat{b}\right),\ell ) & =a^{0}\left(\ell ,k\right)-{\hat{b}}^{H}a^{1}\left(\ell ,k\right)-{\hat{b}}^{T}a^{2}\left(\ell ,k\right)+\mathrm{tr}\left(\left(\hat{b}{\hat{b}}^{H}\right)A\left(\ell ,k\right)\right). \end{array}$$

**Figure 2:**
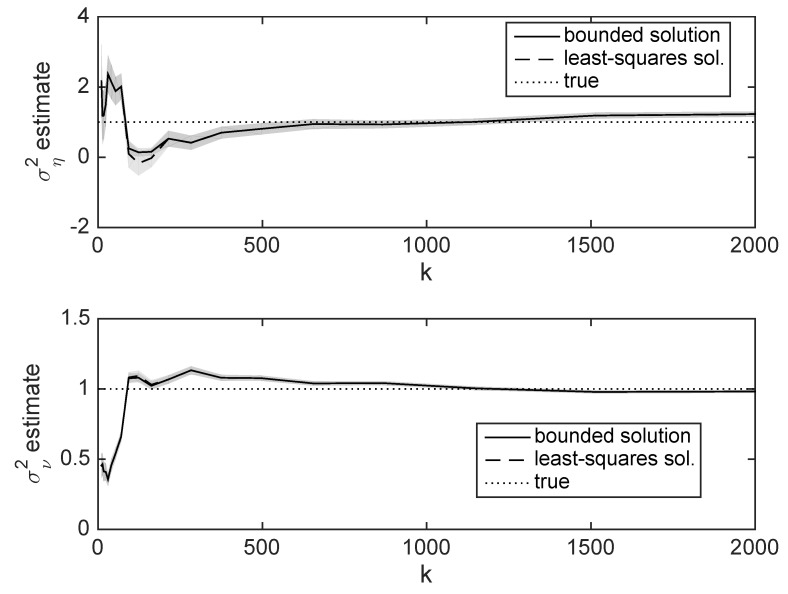
Example of estimating the variances $\sigma_{\nu }^{2}$ (**top plot**) and $\sigma_{\eta }^{2}$ (**bottom plot**), using true b. *k* indicates the number of points used in the estimates. Shading indicates standard deviation of the estimates over 50 iterations. Bounded solution is computed using CVX to avoid negative estimates.

Figure 2 shows the result of this estimation in an example with p=3 and d=5. The mean and standard deviation over 50 independent runs is shown, for up 2000 samples averaged to produce Σ(b). The true value of b is used. The least-squares solution to (18) can produce variance estimates for $\sigma_{\eta }^{2}$ which are negative, which is nonphysical. Solutions constrained so that the variances are constrained to the range [0.1,5] were also computed using CVX [113]. From these results, about 500 samples are needed before the variance estimate converges to a value somewhat close to the true value.

## 4. Vector Autoregressive Formulation

In this section we extend the results of the previous section to vector autoregressive random processes in noise, where at each time vector of length *K* is produced, and the coefficients are K×K matrices. In the interest of generality, a constant offset v is also included. The noisy observations can be written as

$$\begin{array}{cc} y(m) & =\xi \left(m\right)+\nu \left(m\right)=v+\sum_{i=1}^{p}B_{i}\xi \left(m-i\right)+\eta \left(m\right)+\nu \left(m\right). \end{array}$$

As in the scalar case, the noisy observations can be written as

$$y\left(m\right)=v+\sum_{i=1}^{p}B_{i}y\left(m-i\right)+w\left(m\right),$$

where

$$w\left(m\right)=\eta \left(m\right)+\nu \left(m\right)-\sum_{i=1}^{p}B_{i}\nu \left(m-i\right).$$

Following the usual practice [15], this is vectorized to obtain a vector of unknown parameters as follows.

$$y\left(m\right)=\left[\begin{array}{cccc} v & B_{1} & \cdots & B_{p} \end{array}\right]\left[\begin{array}{c} 1\\ y(m-1)\\ \vdots \\ y(m-p) \end{array}\right]+w\left(m\right).$$

Applying the vec operator ([114], Chapter 9) we obtain

$$\begin{array}{cc} y(m) & =\left({\left[\begin{array}{c} 1\\ y(m-1)\\ \vdots \\ y(m-p) \end{array}\right]}^{T}⊗I_{K}\right)\mathrm{vec}\left(\left[\begin{array}{cccc} v & B_{1} & \cdots & B_{p} \end{array}\right]\right)+w\left(m\right). \end{array}$$

Let

$$b=\mathrm{vec}\left(\left[\begin{array}{cccc} v & B_{1} & \cdots & B_{p} \end{array}\right]\right)\in C^{(K^{2}p+K)\times 1}$$

and

$$Y\left(m\right)=\left({\left[\begin{array}{c} 1\\ y(m-1)\\ \vdots \\ y(t-p) \end{array}\right]}^{T}⊗I_{K}\right)\in C^{K\times (K^{2}p+K)}.$$

Then

y(m)=Y(m)b+w(m).

The noise structure for a single vector can be written as

$$w\left(m\right)=\left[\begin{array}{cccccc} I & I & -B_{1} & -B_{2} & \cdots & -B_{p} \end{array}\right]\left[\begin{array}{c} \eta (m)\\ \nu (m)\\ \nu (m-1)\\ \nu (m-2)\\ \vdots \\ \nu (m-p) \end{array}\right].$$

Then

$$E\left[w\left(m\right)w^{H}\left(m\right)\right]=2(\sigma_{\eta }^{2}I+\sigma_{\nu }^{2}\left(I+\sum_{i=1}^{p}B_{i}B_{i}^{H}\right)\overset{▵}{=}R_{w}\left(0\right).$$

We also find

$$E\left[w\left(m\right)w^{H}\left(m+\ell \right)\right]=2\sigma_{\nu }^{2}\left(-B_{\ell }^{H}+\sum_{i=1}^{p-\ell }B_{i}B_{i+\ell }^{H}\right)\overset{▵}{=}R_{w}\left(\ell \right).$$

As in the scalar case, stack up multiple observations so that the correlation structure may be exploited:

$$\left[\begin{array}{c} y(m)\\ y(m-1)\\ \vdots \\ y(m-d+1) \end{array}\right]=\left[\begin{array}{c} Y(m)\\ Y(m-1)\\ \vdots \\ Y(m-d+1) \end{array}\right]b+\left[\begin{array}{c} w(m)\\ w(m-1)\\ \vdots \\ w(m-d+1) \end{array}\right].$$

Write this as

y(m)=Y(m)b+w(m).

The correlation structure of the stacked noise vector is

$$\begin{array}{c} E[w\left(m\right)w{\left(m\right)}^{H}]=\\ \;\left[\begin{array}{cccc} R_{w}\left(0\right) & R_{w}\left(-1\right) & \cdots & R_{w}\left(-d+1\right)\\ R_{w}\left(1\right) & R_{w}\left(0\right) & \cdots & R_{w}\left(-d+2\right)\\ \vdots \\ R_{w}\left(d-1\right) & R_{w}\left(d-2\right) & \cdots & R_{w}\left(0\right) \end{array}\right]\\ \;\overset{▵}{=}R\left(b\right). \end{array}$$

Let y(m),y(m+s),…,y(m+sk) and Y(m),Y(m+s),…,Y(m+sk) be a sequence of vectors and matrices where the vector samples are skipped by *s* samples at each step, and for convenience denote these as $y_{1},y_{2},\ldots ,y_{k}$ and $Y_{1},Y_{2},\ldots ,Y_{k}$. Following the method of Section 2, the estimate

$${\hat{b}}_{k}=\arg\max_{b}\sum_{i=1}^{k}\lambda^{k-i}{∥y_{i}-Y_{i}b∥}_{R{\left(b\right)}^{-1}}$$

is determined by the solution to the normal equations

$$\begin{array}{c} {\left(\sum_{i=0}^{k}\lambda^{k-i}Y_{i}^{H}R{\left(\hat{b}\right)}^{-1}Y_{i}\right)}^{-1}b=\left(\sum_{i=0}^{k}\lambda^{k-i}Y_{i}^{H}R_{w}{\left(b\right)}^{-1}.y_{i}\right) \end{array}$$

With the appropriate use of vectorized data, Algorithm 1 can be used for the VAR as well.

## 5. Some Results

Several test cases were examined to determine the performance of the ICWARE approach; these are summarized in Table 1, where the pole locations are given in polar form $\rho e^{j\theta }$.

**Figure 3:**
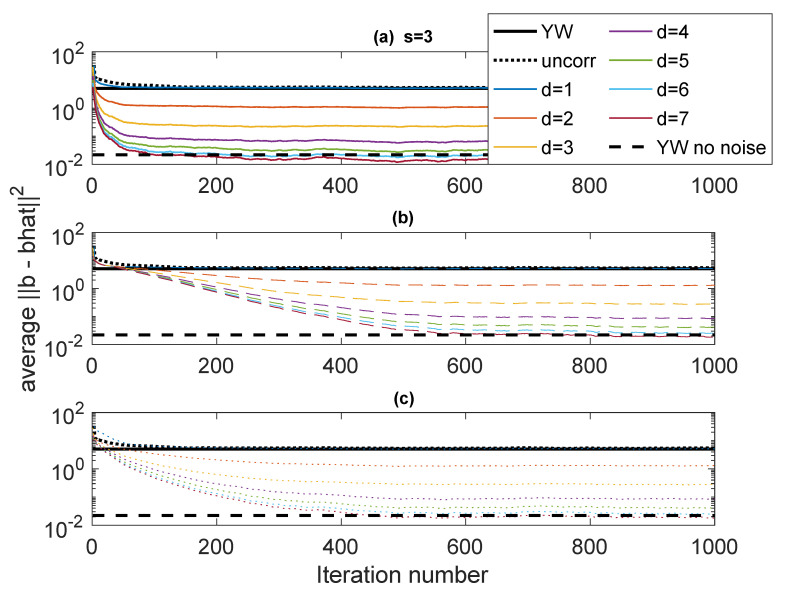
Case 1: Error performance fo different values of *d* with s=3. (**a**) Solid: True $R_{b}\left(b\right)$; (**b**) Dashed: $R_{b}\left(\hat{b}\right)$ estimated from $\hat{b}$ and correct variances; (**c**) Dotted: $R_{b}\left(b\right)$ estimated from $\hat{b}$ and fixed incorrect variances. Comparison: Black dotted: conventional least-squares; Solid black: Yule-Walker (YW) with noisy observations; Dashed black: NW with noise-free observations.

The first example, designated Case 1, is a system with p=3 having poles at $\rho e^{j\theta_{1}},\rho e^{j\theta_{2}},\rho e^{j\theta_{3}}$, with ρ=0.95 and $\left(\theta_{1},\theta_{2},\theta_{3}\right)=\left(0.65,0.7,0.75\right)$. resulting in a fairly narrowband complex AR signal. The noise variances are $\sigma_{\eta }^{2}=1$, and $\sigma_{\nu }^{2}=1$. This case is used to explore some of variations in performance as parameters of the algorithm are varied. Figure 3 shows $∥b-\hat{b}{∥}_{2}^{2}$ as a function of iteration using various forms of $R_{b}\left(b\right)$ and values of *d* (the stack height) from 1 to 7. The “skip” is set to s=3. The results are obtained by averaging the results of 50 independent runs. In these plots, “Iteration number” refers to the number of blocks of data used in an online scheme, each iteration of the algorithm corresponding to one data block. The results are compared with $∥b-\hat{b}{∥}_{2}^{2}$ for YW with noisy measurements and noise-free measurements and scalar AR estimation. The autocorrelation values for the YW method are computed using 30000 points. The Burg method was also used, but with this many points there is little difference between Burg and conventional YW. The scalar AR estimation (black dotted line) converges to the YW performance, as does the ICWARE with d=1, as expected.

In Figure 3, three different ways of determining $R_{b}\left(b\right)$ are used. The solid lines (subplot (a)) use the true $R_{b}\left(b\right)$. This is, of course, unavailable in practice, but these plots serve as a basis for comparison against real algorithms. The dashed lines (subplot (b)) use an identity matrix for $R_{b}\left(b\right)$ for the first 30 iterations (to establish an estimate of b), following which $R_{b}\left(\hat{b}\right)$ is computed using estimated b and the correct variances $\sigma_{\nu }^{2}$ and $\sigma_{\epsilon }^{2}$. The dotted lines (subplot (c)) are similar, except that fixed (but wrong) values of $\sigma_{\nu }^{2}=5$ and $\sigma_{\epsilon }^{2}=5$ are used in the computation of $R_{b}\left(\hat{b}\right)$. It is clear that as *d* increases, the performance significantly improves, especially for the first few values of *d*. Interestingly, for d=7, the error performance is on the order of the error in the noise-free YW.

The improvements of the new method after 1000 iterations (denoted by $∥b-{\hat{b}}_{k}{∥}_{d}^{2}$) compared with the YW result $∥b-{\hat{b}}_{YW}{∥}_{2}^{2}$ tabulated in Table 2 for s=2,3 and 4. There is little difference between s=2 and s=3, but when s=4, that is, s>p, the performance declines.

**Figure 4:**
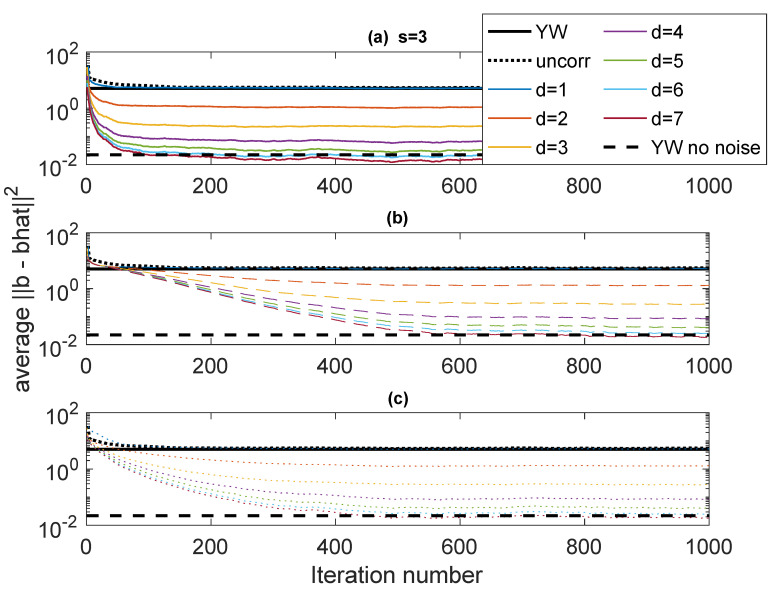
Case 1: Error performance for different values of *s* with d=7. (**a**) Solid: True $R_{b}\left(b\right)$; (**b**) Dashed: $R_{b}\left(b\right)$ estimated from $\hat{b}$ and variances; (**c**) Dotted: $R_{b}\left(b\right)$ estimated from $\hat{b}$ and fixed incorrect variances. Comparison: Black dotted: conventional least-squares; Solid black: Yule-Walker (YW) with noisy observations; Dashed black: NW with noise-free observations.

Figure 4 shows the influence of the skip *s* on the performance, with the same methods of estimating $R_{b}\left(b\right)$ as shown in Figure 3. For a fixed value of d=7, the error curves for different values of *s* are shown. Larger *s* for s≤p does improve the performance, but only slightly.

(a) Solid: True $R_{b}\left(b\right)$; (b) Dashed: $R_{b}\left(\hat{b}\right)$ estimated from $\hat{b}$ and correct variances; (c) Dotted: $R_{b}\left(b\right)$ estimated from $\hat{b}$ and fixed incorrect variances.

**Figure 5:**
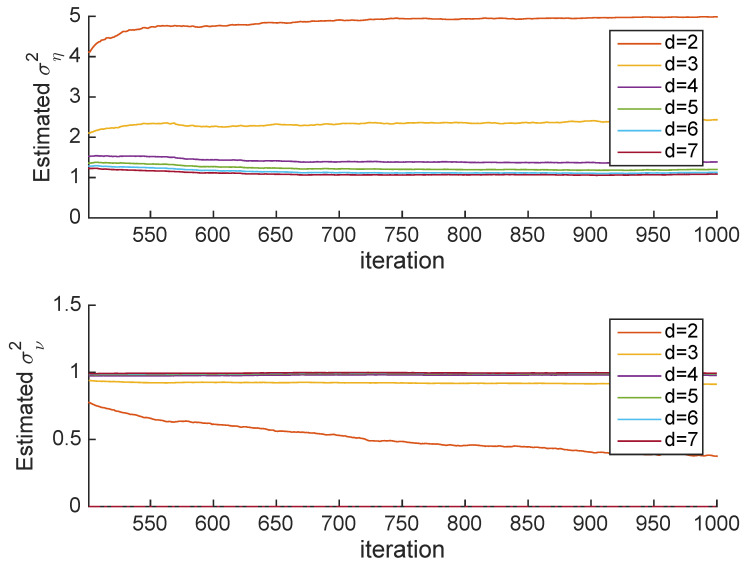
Case 1: Estimated variances for different values of *d*. **Top**: Estimated $\sigma_{\eta }^{2}$; **Bottom**: Estimated $\sigma_{\nu }^{2}$.

Figure 5 shows estimates of the variances $\sigma_{\nu }^{2}$ and $\sigma_{\eta }^{2}$ for different values of *d*. Obviously we need d>2 (in order to have two equations). However, when d=2 there is a very large variance. By the point d≥4, the variance estimates are very close to the true variances.

**Figure 6:**
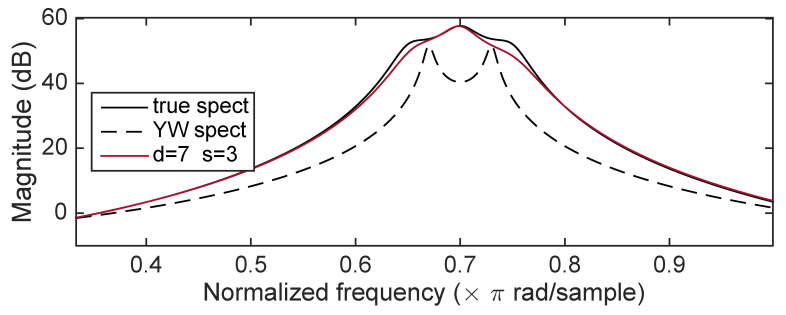
Case 1: Comparison of ICWARE estimated spectrum with true spectrum, and the YW estimated spectrum.

Figure 6 shows the magnitude frequency response for the true spectrum, the YW spectrum, and the spectrum computed from $\hat{b}$ obtained using d=7 and d=3 after convergence. (The spectrum is not an average, but only a single outcome.) The ICWARE estimated spectrum is very close to the true spectrum while the YW spectrum has considerable bias, exhibiting strong peaks not present in the true spectrum.

**Figure 7:**
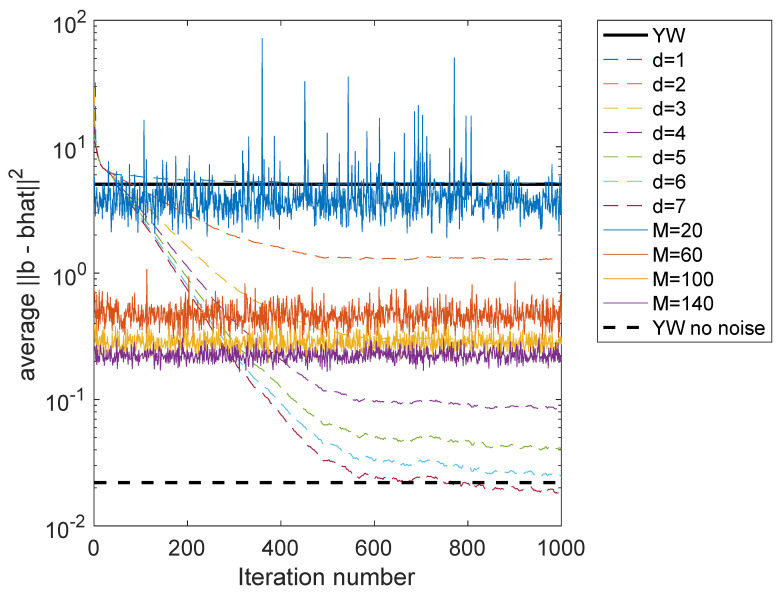
Case 1: Comparison of new method with total least squares (TLS) solutions for different TLS sizes. Dashed: ICWARE for different values of *d*; Solid: TLS for different matrix sizes.

As a point of comparison, TLS is used to estimate the AR parameters. In TLS, the equation

(19) $$\begin{array}{cc} \; & \left[\begin{array}{cccc} y(m) & y(m-1) & \cdots & y(m-p+1)\\ y(m+1) & y(m) & \cdots & y(m-p+2)\\ \vdots \\ y(m+M) & y(m+M-1) & \cdots & y(m+M-p) \end{array}\right]\left[\begin{array}{c} b_{1}\\ b_{2}\\ \vdots \\ b_{p} \end{array}\right]=\left[\begin{array}{c} y(m+1)\\ y(m+2)\\ \vdots \\ y(m+M+1) \end{array}\right]+\left[\begin{array}{c} \nu (m+1)\\ \nu (m+2)\\ \vdots \\ \nu (m+M+1) \end{array}\right] \end{array}$$

(for some *m* and *M*) is perturbed in both the matrix on the left and the vector of observations on the right. TLS can be computed using the singular value decomposition (SVD), making it computationally complex. It also makes it more difficult to create algorithms which track changing parameters, and, as shown below, gives inferior parameter estimates than the method presented here. Figure 7 shows the error for $\hat{b}$ computed using TLS. The parameter *M* is the height of the linear system that is solved using the TLS method described in Reference [114]. The values M∈{20,60,100,140} are examined. At each iteration, a different set of points are used in the TLS equations, each of which is computed using a (relatively complicated) singular value decomposition. Even with 20 rows in the TLS equations, performance only roughly comparable to the YW equations is obtained. For 60 or more equations, the improvement of the performance quickly saturates, so that 60 or 140 perform rather comparably. The computational complexity is rather high. For each solution (at each iteration), the SVD of a M×3 matrix is computed. Despite the computational complexity, the ICWARE method performs superior to the TLS for d≥4. (The method of estimating $R_{b}\left(b\right)$ in is the same as used for Figure 3b.)

**Figure 8:**
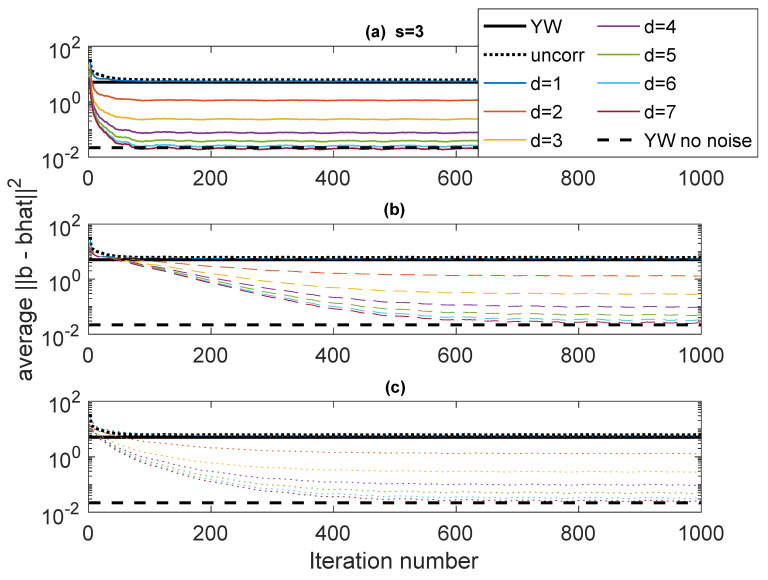
Case 1: Error performance of 10 repetitions on blocks of length 100. (**a**) Solid: True $R_{b}\left(b\right)$; (**b**) Dashed: $R_{b}\left(b\right)$ estimated from $\hat{b}$ and variances; (**c**) Dotted: $R_{b}\left(b\right)$ estimated from $\hat{b}$ and fixed incorrect variances. Comparison: Black dotted: conventional least-squares; Solid black: Yule-Walker (YW) with noisy observations; Dashed black: NW with noise-free observations.

**Figure 9:**
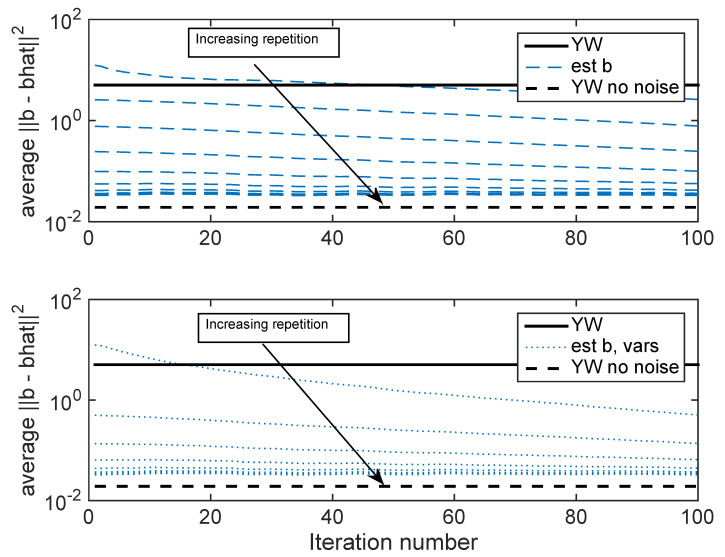
Performance on 10 repetitions on blocks of length 100, with the results folded for each iteration. **Top**: Estimated $R_{b}\left(\hat{b}\right)$ using $\hat{b}$ and true variances; **Bottom**: Estimated $R_{b}\left(\hat{b}\right)$ using $\hat{b}$ and fixed variances.

The results presented to this point take 1000 blocks of data. A consideration is whether it is possible to iteratively re-use data so that less data is required, but the reduced error from the ICWARE algorithm can be obtained. In Figure 8, k=100 blocks of data are employed, and the processing is repeated 10 times on each block. Figure 9 shows the error as a result of each iteration. Each line in the plot corresponds to one pass through the data. The top plot uses $R_{b}\left(\hat{b}\right)$ estimated using $\hat{b}$ and true variances; the bottom plot uses $R_{b}\left(\hat{b}\right)$ using $\hat{b}$ and fixed variances. These figures show that iterating over the data provides essentially the same performance as longer data.

**Figure 10:**
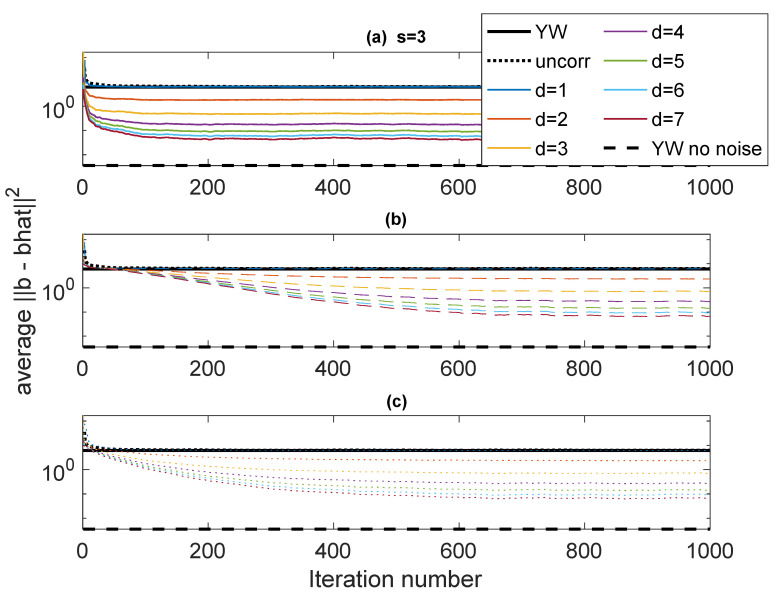
Case 2: Error performance for different values of *d* with s=3. (**a**) Solid: True $R_{b}\left(b\right)$; (**b**) Dashed: $R_{b}\left(b\right)$ estimated from $\hat{b}$ and variances; (**c**) Dotted: $R_{b}\left(b\right)$ estimated from $\hat{b}$ and fixed incorrect variances. Comparison: Black dotted: conventional least-squares; Solid black: Yule-Walker (YW) with noisy observations; Dashed black: NW with noise-free observations.

**Figure 11:**
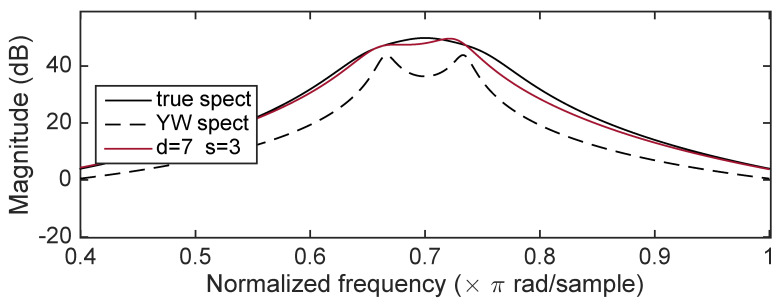
Case 2: Comparison of ICWARE estimated spectrum with true spectrum, and the YW estimated spectrum.

The next example, designated Case 2, is also a third order system with poles at the same angles as Case 1, but with ρ=0.9. The following figures show examples of the same sort of results shown for Case 1. In Figure 10 the various methods are compared. In this case, however, the minimum error does not reach as low as the noise-free YW case. Figure 11 shows the resulting spectrum, again showing that the ICWARE spectrum is closer than the YW spectrum.

**Figure 12:**
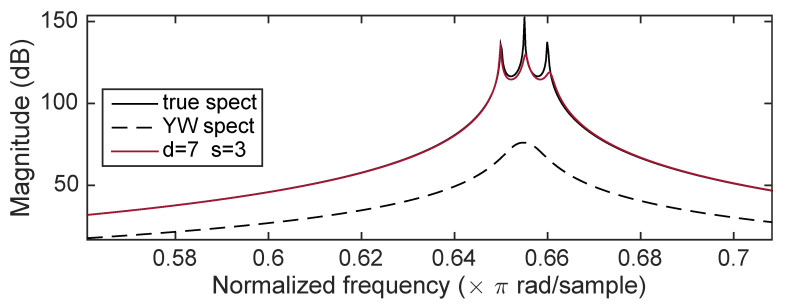
Case 3: Comparison of ICWARE estimated spectrum with true spectrum, and the YW estimated spectrum.

Case 3 involves a system having poles at ρ=1—that is, pure sinusoidal signals—and angles $\left(\theta_{1},\theta_{2},\theta_{3}\right)=\left(0.65,0.655,0.66\right)$. The estimated spectra are shown in Figure 12. The spectral peaks are clearly evident in the ICWARE estimate.

**Figure 13:**
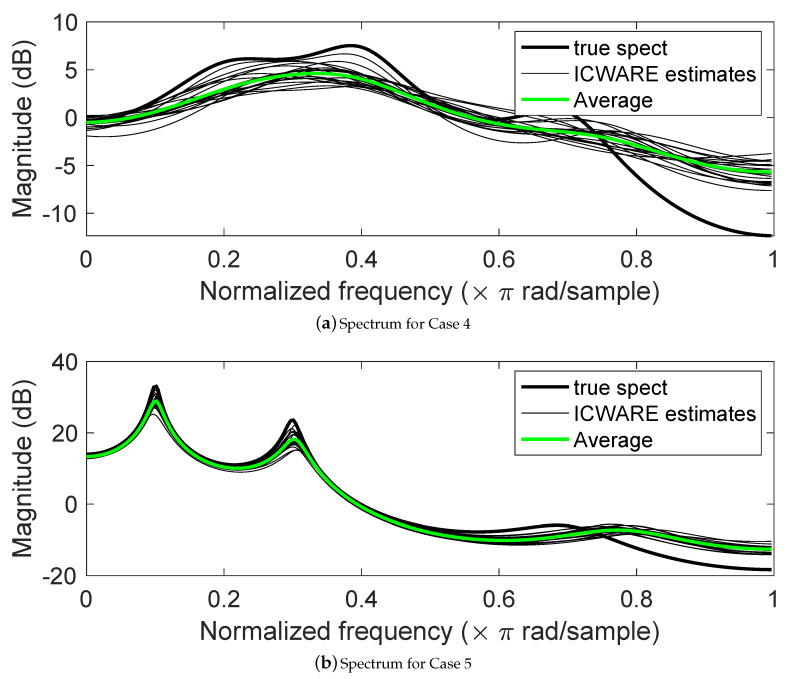

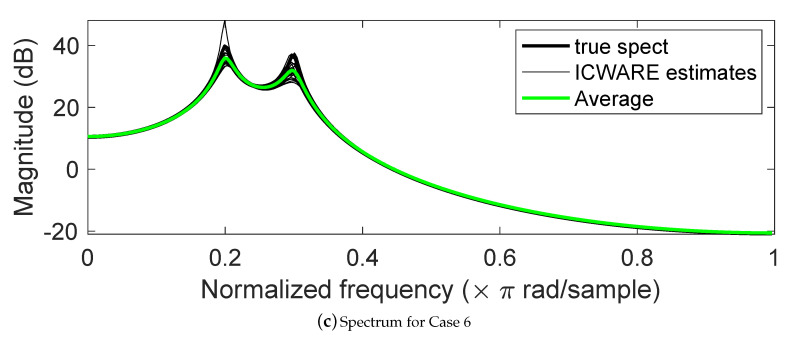
Spectral estimation results for examples from Reference [104]. (**a**) Case 4; (**b**) Case 5; (**c**) Case 6. $\sigma_{\eta }^{2}=1$.

As another comparison, ICWARE spectral estimates are compared to the spectra from dual Kalman filters using the examples from Reference [104], shown as Cases 4,5, and 6 in Table 1. Figure 13 shows the results. Spectra for twenty realizations are plotted, along with the true spectrum and the mean. The values of d=p+3 and s=3 were selected, since simulations for Case 1 (above) suggest these are reasonable values. Comparison of these results with Figures 3, 4, and 6 of Reference [104] shows fairly comparable performance. When the poles are not near the unit circle (Case 4), the ICWARE estimate tend to have smoother spectra (estimated poles with smaller magnitude). When the poles are nearer the units circle (Case 5, Case 6), ICWARE seems to do a comparable job at capturing the peaks, and does somewhat better at representing the high-frequency dropoff of the spectrum. In all of these simulations, an estimated $R_{b}\left(b\right)$ was employed.

## 6. Summary and Conclusions

In this paper, we have shown that accounting for observation noise added to a pure AR(*p*) process results in noise which is correlated across lags. This makes it expedient to employ stacked observation vectors, and estimating the parameters in a vector sense. This results in an algorithm that is essentially a vector RLS adaptive filter. Several different methods were described to obtain information about the correlation matrix $R_{w}\left(b\right)$. Also, estimating the variances of the AR and observation noise was described.

It was shown by simulation that the method can be applied repetitively to the same block of data, providing accurate results with data of moderate length.

The improvement of the technique compared with Yule-Walker is a function of the depth *d* to which the observations are stacked. Values even greater than *p* continue to yield improvement. The improvement relative to YW also is system dependent. Based on simulations, a depth d=p+3, where *p* is the order of the system, seems a reasonable choice.

Comparisons were made against a dual Kalman filter approach, with comparable or slightly superior results.

## Figures and Tables

**Table 1:** Parameters for test cases.

Case	Order	Pole Locations
1	3	$0.95e^{j2\pi 0.65}$, $0.95e^{j2\pi 0.7}$, $0.95e^{j2\pi 0.75}$
2	3	$0.9e^{j2\pi 0.65}$, $0.9e^{j2\pi 0.7}$, $0.9e^{j2\pi 0.75}$
3	3	$e^{j2\pi 0.65}$, $e^{j2\pi 0.7}$, $e^{j2\pi 0.75}$
4	6	$0.75e^{\pm j2\pi 0.1}$, $0.8e^{\pm j2\pi 0.2}$, $0.85e^{\pm j2\pi 0.35}$
5	6	$0.98e^{\pm j2\pi 0.05}$, $0.97e^{\pm j2\pi 0.15}$, $0.8e^{\pm j2\pi 0.35}$
6	6	$0.98e^{\pm j2\pi 0.1}$, $0.97e^{\pm j2\pi 0.1}$, $0.98e^{\pm j2\pi 0.15}$

**Table 2:** Comparison of $∥b-{\hat{b}}_{k}{∥}^{2}$ computed for d=1,…,7 with $∥b-{\hat{b}}_{YW}{∥}^{2}$ for YW.

*d*	$10\log_{10}∥b-{\hat{b}}_{k}{∥}_{2}^{2}/{∥b-{\hat{b}}_{\mathrm{YW}}∥}^{2}$		
	s=2	s=3	s=4
2	−5.9	−5.8	11.1
3	−12.4	−12.5	4.5
4	−17.5	−17.5	−0.6
5	−20.6	−20.6	−3.9
6	−22.6	−22.7	−5.9
7	−23.9	−24.0	−7.2

## Appendix A. Maximizing The Log Likelihood Function for Estimating Variances

In this section we present details on the computation of the derivative of the likelihood (12) with respect to $\sigma_{\nu }^{2}$. We note that

$$\frac{\partial }{\partial \sigma_{\eta }^{2}}R_{w}\left(b\right)=2I\;\;\frac{\partial }{\partial \sigma_{\nu }^{2}}R_{w}\left(b\right)=2\sigma_{\nu }^{2}B\left(b\right).$$

The derivative of the second term of (12) is

(A1) $$\begin{array}{cc} \; & \frac{\partial }{\partial \sigma_{\eta }^{2}}\log\det R_{w}\left(b\right)=\sum_{i,j}{\left(\frac{\partial }{\partial R_{w}\left(b\right)}\log\det R_{w}\left(b\right)\right)}_{i,j}{\left(\frac{\partial }{\partial \sigma_{\eta }^{2}}R_{w}\left(b\right)\right)}_{i,j}\\ \; & =\sum_{i,j}{\left(R_{w}{\left(b\right)}^{-1}\right)}_{i,j}{\left(I\right)}_{i,j}=\mathrm{tr}\left(R_{w}{\left(b\right)}^{-1}\right). \end{array}$$

The summation term of (12) can be written as

$$\begin{array}{c} \sum_{i=1}^{k}{\left(y_{i}-Y_{i}b\right)}^{H}R_{w}{\left(b\right)}^{-1}\left(y_{i}-Y_{i}b\right)=\mathrm{tr}\left(\sum_{i=1}^{k}{\left(y_{i}-Y_{i}b\right)}^{H}R_{w}{\left(b\right)}^{-1}\left(y_{i}-Y_{i}b\right)\right)\\ \;=k\mathrm{tr}\left(R_{w}{\left(b\right)}^{-1}\Sigma \left(b\right)\right) \end{array}$$

so that the derivative is

(A2) $$\begin{array}{cc} \; & \frac{\partial }{\partial \sigma_{\eta }^{2}}\sum_{i=1}^{k}{\left(y_{i}-Y_{i}b\right)}^{H}R_{w}{\left(b\right)}^{-1}\left(y_{i}-Y_{i}b\right)=\\ \; & k\sum_{i,j}{\left(\frac{\partial }{\partial R_{w}\left(b\right)}\mathrm{tr}\left(R_{w}{\left(b\right)}^{-1}\Sigma \left(b\right)\right)\right)}_{i,j}{\left(\frac{\partial }{\partial \sigma_{\eta }^{2}}R_{w}\left(b\right)\right)}_{i,j}\\ \; & \;=k\sum_{i,j}{\left(-R_{w}{\left(b\right)}^{-1}\Sigma \left(b\right)R_{w}{\left(b\right)}^{-1}\right)}^{H}{\left(I\right)}_{i,j}\\ \; & \;=-\mathrm{tr}(R_{w}{\left(b\right)}^{-1}\Sigma \left(b\right)R_{w}{\left(b\right)}^{-1}). \end{array}$$

Combining (A1) and (A2) gives (14).

## References

[1] Makhoul J. (1973). Spectral analysis of speech by linear prediction. IEEE Trans. Audio Electroacoust..

[2] Lim J., Oppenheim A. (1978). All-pole modeling of degraded speech. IEEE Trans. Signal Process..

[3] Lim J., Oppenheim A. (1979). Enhancement and bandwidth compression of noisy speech. Proc. IEEE Inst. Electr. Electron. Eng..

[4] Wrench A., Cowan C. A new approach to noise-robust LPC. Proceedings of the IEEE International Conference on Acoustics, Speech, and Signal Processing, ICASSP ’87.

[5] Kay S. (1988). Modern Spectral Estimation: Theory and Application.

[6] Deller J.R., Proakis J.G., Hansen J.H.L. (1993). Discrete–Time Processing of Speech Signals.

[7] Ramamurthy K.N., Spanias A.S. (2009). Matlab Software for The Code Excited Linear Prediction Algorithm: The Federal Standard 1016.

[8] de Prony B.R. (1795). Essai Expérimental et Analytique: Sur les Lois de la Dilatabilité de Fluides Élastiques et sur Celles de la Force Exapnsive de la Vapeur de l’eau et de la Vapeur de l’alcool, à Différentes Températures. J. l’École Polytech..

[9] Ulrich T., Clayton R. (1976). Time series modeling and maximum entropy. Phys. Earth Planetary Interiors.

[10] Capon J. (1969). High-Resolution Frequency-Wavenumber Spectrum Analysis. Proc. IEEE.

[11] Burg J. (1975). Maximum entropy spectral analysis. Ph.D. Thesis.

[12] Marple L. High Resolution Autoregressive Spectrum Analysis Using Noise Power Cancellation. Proceedings of the IEEE International Conference on Acoustics, Speech, and Signal Processing, ICASSP ’78.

[13] Marple L. (1980). A New Autoregressive Spectrum Analysis Algorithm. IEEE Trans. ASSP.

[14] Nuttall A. (1976). Spectral Analysis of a Univariate Process with Bad Data Points, Via Maximum Entropy and Linear Predictive Techniques.

[15] Helmut Lütkepohl (2005). New Introduction fo Multiple Time Series Analysis.

[16] Guler I., Kiymik M., Akin M., Alkan A. (2001). AR spectral analysis of EEG signals by using maximum likelihood estimation. Comput. Biol. Med..

[17] Baddour K., Beaulieu N. (2005). Autoregressive modeling for fading channel simulation. IEEE Trans. Wireless Comm..

[18] Box G.E.P., Jenkins G.M. (1978). Time Series Analysis: Forecasting and Control.

[19] Makhoul J. (1975). Linear Prediction: A Tutorial Review. Proc. IEEE.

[20] Sayed A.H. (2003). Fundamentals of Adaptive Filtering.

[21] Anderson B.D.O., Moore J.B. (1979). Optimal Filtering.

[22] Kay S. (1979). The Effects of Noise on the Autoregressive Spectral Estimator. IEEE Trans. Acoust. Speech Signal Process..

[23] Lacoss R.T. (1971). Data adaptive spectral analysis methods. Geophysics.

[24] Tufts D., Kumaresan R. (1980). Improved spectral resolution. Proc. IEEE.

[25] Tufts D., Kumaresan R. Improved spectral resolution II. Proceedings of the IEEE International Conference on Acoustics, Speech, and Signal Processing, ICASSP ’80.

[26] Kumaresan R., Tufts D. (1980). Improved spectral resolution III: Efficient realization. Proc. IEEE.

[27] Kumaresan R., Tufts D. Accurate parameter estimation of noisy speech-like signals. Proceedings of the Acoustics, Speech, and Signal Processing, IEEE International Conference on ICASSP ’82.

[28] Kumaresan R., Tufts D. (1982). Estimating the parameters of exponentially damped sinusoids and pole-zero modeling in noise. IEEE Trans. Acoust. Speech Signal Process..

[29] Tufts D., Kumaresan R. (1982). Estimation of frequencies of multiple sinusoids: Making linear prediction perform like maximum likelihood. Proc. IEEE.

[30] Kumaresan R., Tufts D., Scharf L. (1984). A Prony method for noisy data: Choosing the signal components and selecting the order in exponential signal models. Proc. IEEE.

[31] Cadzow J. (1980). High Performance Spectral Estimation: A New ARMA Method. IEEE Trans. Acoust. Speech Signal Process..

[32] Zheng W.X. On TLS estimation of autoregressive signals with noisy measurements. Proceedings of the SSeventh International Symposium on Signal Processing and Its Applications.

[33] Huffel S.V., Vandewalle J. The Use of Total Linear Least Squares Technique for Identification and Parameter Estimation. Proceedings of the IFAC/IFORS Symposium on Identification and Parameter Estimation.

[34] van Huffel S.J., Vandewalle S.J., Haegemans A. (1987). An Efficient and Reliable Algorithm for Computing the Singular Subspace of a Matrix, Associated with Its Smallest Singular Values. J. Comput. Appl. Math..

[35] Huffel S.V., Vandewalle J. (1988). The Partial Total Least Squares Algorithm. J. Comput. Appl. Math..

[36] Zheng W. Identification of Autoregressive Signals Observed in Noise. Proceedings of the American Control Conference.

[37] Zheng W.X. An efficient algorithm for parameter estimation of noisy AR processes. Proceedings of the 1997 IEEE International Symposium on Circuits and Systems, ISCAS ’97.

[38] Zheng W. (1997). Estimation of autoregressive signals from noisy measurements. IEEE Proc. Vis. Image Signal Process..

[39] Zheng W.X. Unbiased identification of autoregressive signals observed in colored noise. Proceedings of the 1998 IEEE International Conference on Acoustics, Speech and Signal Processing.

[40] Zheng W.X. Adaptive parameter estimation of autoregressive signals from noisy observations. Proceedings of the ICSP ’98. 1998 Fourth International Conference on Signal Processing Proceedings.

[41] Zheng W.X. On implementation of a least-squares based algorithm for noisy autoregressive signals. Proceedings of the ISCAS ’98, 1998 IEEE International Symposium on Circuits and Systems.

[42] Zheng W.X. (1999). A least-squares based method for autoregressive signals in the presence of noise. IEEE Trans. Circuits Syst. II Analog Digit. Signal Process..

[43] Zheng W.X. Adaptive linear prediction of autoregressive models in the presence of noise. Proceedings of the WCCC-ICSP 2000, 5th International Conference on Signal Processing Proceedings.

[44] Zheng W.X. (2000). Autoregressive parameter estimation from noisy data. IEEE Trans. Circuits Syst. II Analog Digit. Signal Process..

[45] Zheng W.X. (2000). Estimation of the parameters of autoregressive signals from colored noise-corrupted measurements. IEEE Signal Process. Lett..

[46] Zheng W.X. A fast convergent algorithm for identification of noisy autoregressive signals. Proceedings of the ISCAS 2000 Geneva. The 2000 IEEE International Symposium on Circuits and Systems.

[47] Zheng W.X. A new estimation algorithm for AR signals measured in noise. Proceedings of the 2002 6th International Conference on Signal Processing.

[48] Zheng W.X. An alternative method for noisy autoregressive signal estimation. Proceedings of the ISCAS 2002. IEEE International Symposium on Circuits and Systems.

[49] Zheng W.X. On unbiased parameter estimation of autoregressive signals observed in noise. Proceedings of the 2003 International Symposium on Circuits and Systems, ISCAS ’03.

[50] Zheng W.X. Fast adaptive identification of autoregressive signals subject to noise. Proceedings of the 2004 International Symposium on Circuits and Systems, ISCAS ’04.

[51] Zheng W.X. An efficient method for estimation of autoregressive signals in noise. Proceedings of the 2005 IEEE International Symposium on Circuits and Systems, ISCAS 2005.

[52] Zheng W.X. A new look at parameter estimation of autoregressive signals from noisy observations. Proceedings of the 2006 IEEE International Symposium on Circuits and Systems, ISCAS 2006.

[53] Zheng W.X. (2006). On Estimation of Autoregressive Signals in the Presence of Noise. IEEE Trans. Circuits Syst. II Express Briefs.

[54] Zheng W.X. An Efficient Method for Estimation of Autoregressive Signals Subject to Colored Noise. Proceedings of the 2007 IEEE International Symposium on Circuits and Systems, ISCAS 2007.

[55] Xia Y., Zheng W.X. On unbiased identification of autoregressive signals with noisy measurements. Proceedings of the 2015 IEEE International Symposium on Circuits and Systems (ISCAS).

[56] Arablouei R., Dogancay K., Adali T. Unbiased RLS identification of errors-in-variables models in the presence of correlated noise. Proceedings of the 2014 22nd European Signal Processing Conference (EUSIPCO).

[57] Ai-guo W., Fan Y., Yang-Yang Q. Bias compensation based recursive least squares identification for equation error models with colored noises. Proceedings of the 2014 33rd Chinese Control Conference (CCC).

[58] Arablouei R., Dogancay K., Adali T. (2014). Unbiased Recursive Least-Squares Estimation Utilizing Dichotomous Coordinate-Descent Iterations. IEEE Trans. Signal Process..

[59] Yong Z., Hai T., Zhaojing Z. A modified bias compensation method for output error systems with colored noises. Proceedings of the 2011 30th Chinese Control Conference (CCC).

[60] Wu A.G., Qian Y.Y., Wu W.J. (2014). Bias compensation-based recursive least-squares estimation with forgetting factors for output error moving average systems. IET Signal Process..

[61] Arablouei R., Dogancay K., Werner S. (2015). Recursive Total Least-Squares Algorithm Based on Inverse Power Method and Dichotomous Coordinate-Descent Iterations. IEEE Trans. Signal Process..

[62] Tomcik J., Melsa J. Least squares estimation of predictor coefficients from noisy observations. Proceedings of the 1977 IEEE Conference on Decision and Control including the 16th Symposium on Adaptive Processes and A Special Symposium on Fuzzy Set Theory and Applications.

[63] Lee T.S. Identification and spectral estimation of noisy multivariate autoregressive processes. Proceedings of the IEEE International Conference on Acoustics, Speech, and Signal Processing, ICASSP ’81.

[64] Cadzow J.A. (1981). Autoregressive Moving Average Spectral Estimation: A Model Equation Error Procedure. IEEE Trans. Geosci. Remote Sens..

[65] Gingras D. Estimation of the autoregressive parameters from observations of a noise corrupted autoregressive time series. Proceedings of the IEEE International Conference on Acoustics, Speech, and Signal Processing, ICASSP ’82.

[66] Ahmed M. Estimating the parameters of a noisy AR-process by using a bootstrap estimator. Proceedings of the IEEE International Conference on Acoustics, Speech, and Signal Processing, ICASSP ’82.

[67] Tugnait J. (1986). Recursive parameter estimation for noisy autoregressive signals. IEEE Trans. Inform. Theory.

[68] Paliwal K. A noise-compensated long correlation matching method for AR spectral estimation of noisy signals. Proceedings of the IEEE International Conference on Acoustics, Speech, and Signal Processing, ICASSP ’86.

[69] Cernuschi-Frias B., Rogers J. (1988). On the exact maximum likelihood estimation of Gaussian autoregressive processes. IEEE Trans. Acoust. Speech Signal Process..

[70] Gingras D., Masry E. (1988). Autoregressive spectral estimation in additive noise. IEEE Trans. Acoust. Speech Signal Process..

[71] Masry E. (1991). Almost sure convergence analysis of autoregressive spectral estimation in additive noise. IEEE Trans. Inform. Theory.

[72] Deriche M. AR parameter estimation from noisy data using the EM algorithm. Proceedings of the 1994 IEEE International Conference on Acoustics, Speech, and Signal Processing, ICASSP-94.

[73] Lee L.M., Wang H.C. (1996). An extended Levinson-Durbin algorithm for the analysis of noisy autoregressive process. IEEE Signal Process. Lett..

[74] Hasan T., Yahagi T. Accurate noise compensation technique for the identification of multichannel AR processes with noise. Proceedings of the 1996 IEEE International Symposium on Circuits and Systems Connecting the World, ISCAS ’96.

[75] Doblinger G. An adaptive Kalman filter for the enhancement of noisy AR signals. Proceedings of the 1998 IEEE International Symposium on Circuits and Systems, ISCAS ’98.

[76] Hasan T., Hossain J. Multichannel autoregressive spectral estimation from noisy observations. Proceedings of the TENCON 2000.

[77] Hasan T., Ahmed K. A joint technique for autoregressive spectral estimation from noisy observations. Proceedings of the TENCON 2000.

[78] Davila C. (2001). On the noise-compensated Yule-Walker equations. IEEE Trans. Signal Process..

[79] So H. (2001). LMS algorithm for unbiased parameter estimation of noisy autoregressive signals. Electron. Lett..

[80] Jin C.Z., Jia L.J., Yang Z.J., Wada K. On convergence of a BCLS algorithm for noisy autoregressive process estimation. Proceedings of the 41st IEEE Conference on Decision and Control.

[81] Hasan M., Rahim Chowdhury A., Adnan R., Rahman Bhuiyan M., Khan M. A new method for parameter estimation of autoregressive signals in colored noise. Proceedings of the 2002 11th European Signal Processing Conference.

[82] Hasan T., Fattah S., Khan M. (2003). Identification of noisy AR systems using damped sinusoidal model of autocorrelation function. IEEE Signal Process. Lett..

[83] Hasan M., Chowdhury A., Khan M. (2003). Identification of autoregressive signals in colored noise using damped sinusoidal model. IEEE Trans. Circuits Syst. I Fundam. Theory Appl..

[84] Diversi R., Guidorzi R., Soverini U. Identification of ARX models with noisy input and output. Proceedings of the 2007 European Control Conference (ECC).

[85] Fattah S., Zhu W., Ahmad M. Identification of autoregressive moving average systems from noise-corrupted observations. Proceedings of the 2008 Joint 6th International IEEE Northeast Workshop on Circuits and Systems and TAISA Conference, NEWCAS-TAISA 2008.

[86] Fattah S., Zhu W., Ahmad M. A correlation domain algorithm for autoregressive system identification from noisy observations. Proceedings of the 51st Midwest Symposium on Circuits and Systems, MWSCAS 2008.

[87] Babu P., Stoica P., Marzetta T. An IQML type algorithm for AR parameter estimation from noisy covariance sequences. Proceedings of the 2009 17th European Signal Processing Conference.

[88] Qu X., Zhou J., Luo Y. An Advanced Parameter Estimator of Multichannel Autoregressive Signals from Noisy Observations. Proceedings of the 2009 International Conference on Information Engineering and Computer Science, ICIECS 2009.

[89] Weruaga L., Al-Ahmad H. Frequency-selective autoregressive estimation in noise. Proceedings of the 2010 IEEE International Conference on Acoustics Speech and Signal Processing (ICASSP).

[90] Mahmoudi A., Karimi M. Parameter estimation of noisy autoregressive signals. Proceedings of the 2010 18th Iranian Conference on Electrical Engineering (ICEE).

[91] Weruaga L. Two methods for autoregressive estimation in noise. Proceedings of the 2011 IEEE GCC Conference and Exhibition (GCC).

[92] Youcef A., Diversi R., Grivel E. Errors-in-variables identification of noisy moving average models. Proceedings of the 2015 23rd European Signal Processing Conference (EUSIPCO).

[93] Hamilton J.D. (1994). Time Series Analysis.

[94] Kay S. (1983). Recursive Maximum Likelihood Estimation of Autoregressive Processes. IEEE Trans. Acoust. Speech Signal Process..

[95] Evans A., Fischl R. (1973). Optimal least squares time-domain synthesis of recursive digital filters. IEEE Trans. Audio Electroacoust..

[96] Bresler Y., Macovski A. (1986). Exact maximum likelihood parameter estimation of superimposed exponentials in noise. IEEE Trans. Acoust. Speech Signal Process..

[97] Steiglitz K., McBride L. (1965). A technique for the identification of linear systems. IEEE Trans. Autom. Control.

[98] Kumaresan R., Scharf L., Shaw A. (1986). An Algorithm for pole-zero modeling and spectral analysis. IEEE Trans. Acoust. Speech Signal Process..

[99] Anderson J., Giannakis G. Noisy input/output system identification using cumulants and the Steiglitz-McBride algorithm. Proceedings of the 1991 Conference Record of the Twenty-Fifth Asilomar Conference on Signals, Systems and Computers.

[100] Fuller W.A. (1987). Measurement Error Models.

[101] Petitjean J., Grivel E., Bobillet W., Roussilhe P. Multichannel AR parameter estimation from noisy observations as an errors-in-variables issue. Proceedings of the 2008 16th European Signal Processing Conference.

[102] Petitjean J., Diversi R., Grivel E., Guidorzi R., Roussilhe P. Recursive errors-in-variables approach for AR parameter estimation from noisy observations. Application to radar sea clutter rejection. Proceedings of the 2009 IEEE International Conference on Acoustics, Speech and Signal Processing, ICASSP 2009.

[103] Petitjean J., Grivel E., Diversi R., Guidorzi R. A recursive errors-in-variables method for tracking time varying autoregressive parameters from noisy observations. Proceedings of the 2010 18th European Signal Processing Conference.

[104] Labarre D., Grivel E., Berthoumieu Y., Todini E., Najim M. (2006). Consistent estimation of autoregressive parameters from noisy observations based on two interacting Kalman filters. Signal Process..

[105] Jamoos A., Grivel E., Christov N., Najim M. (2009). Estimation of autoregressive fading channels based on two cross-coupled *H*_∞_ filters. Signal Image Video Process. J. Springer.

[106] Jamoos A., Grivel E., Shakarneh N., Abdel-Nour H. (2011). Dual Optimal filters for parameter estimation of a multivariate autoregressive process from noisy observations. IET Signal Processing.

[107] Bugallo M.F., Martino L., Corander J. (2015). Adaptive importance sampling in signal procesing. Digit. Signal Process..

[108] Urteaga I., Djuric P.M. (2017). Sequential estimation of Hidden ARMA Processes by Particle Filtering—Part I. IEEE Trans. Signal Process..

[109] Urteaga I., Djuric P.M. (2017). Sequential estimation of Hidden ARMA Processes by Particle Filtering—Part II. IEEE Trans. Signal Process..

[110] Martino L., Elvira V., Camps-Valls G. (2018). Distributed Particle Metropolis-Hastings Schemes. IEEE Statistical Signal Processing Workshop.

[111] Kay S.M. (1993). Fundamentals of Statistical Signal Processing: Estimation Theory.

[112] Farahmand S., Giannakis G.B. (2012). Robust RLS in the Presence of Correlated Noise Using Outlier Sparsity. IEEE Trans. Signal Process..

[113] Grant M., Boyd S. (2011). CVX: Matlab Software for Disciplined Convex Programming, Version 1.21. http://cvxr.com/cvx.

[114] Moon T.K., Stirling W.C. (2000). Mathematical Methods and Algorithms for Signal Processing.

